# Ca^2+^-Sensor Neurocalcin δ and Hormone ANF Modulate ANF-RGC Activity by Diverse Pathways: Role of the Signaling Helix Domain

**DOI:** 10.3389/fnmol.2018.00430

**Published:** 2018-11-27

**Authors:** Teresa Duda, Alexandre Pertzev, Sarangan Ravichandran, Rameshwar K. Sharma

**Affiliations:** ^1^Research Divisions of Biochemistry and Molecular Biology, The Unit of Regulatory and Molecular Biology, Salus University, Elkins Park, PA, United States; ^2^Advanced Biomedical Computational Sciences Group, Frederick National Laboratory for Cancer Research Sponsored by the National Cancer Institute, Leidos Biomedical Research Inc., Fredrick, MD, United States

**Keywords:** membrane guanylate cyclase, cyclic GMP, atrial natriuretic factor receptor guanylate cyclase, neurocalcin delta, ANF

## Abstract

Prototype member of the membrane guanylate cyclase family, ANF-RGC (Atrial Natriuretic Factor Receptor Guanylate Cyclase), is the physiological signal transducer of two most hypotensive hormones ANF and BNP, and of the intracellular free Ca^2+^. Both the hormonal and the Ca^2+^-modulated signals operate through a common second messenger, cyclic GMP; yet, their operational modes are divergent. The hormonal pathways originate at the extracellular domain of the guanylate cyclase; and through a cascade of structural changes in its successive domains activate the C-terminal catalytic domain (CCD). In contrast, the Ca^2+^ signal operating via its sensor, myristoylated neurocalcin δ both originates and is translated directly at the CCD. Through a detailed sequential deletion and expression analyses, the present study examines the role of the signaling helix domain (SHD) in these two transduction pathways. SHD is a conserved 35-amino acid helical region of the guanylate cyclase, composed of five heptads, each meant to tune and transmit the hormonal signals to the CCD for their translation and generation of cyclic GMP. Its structure is homo-dimeric and the molecular docking analyses point out to the possibility of antiparallel arrangement of the helices. Contrary to the hormonal signaling, SHD has no role in regulation of the Ca^2+^- modulated pathway. The findings establish and define in molecular terms the presence of two distinct non-overlapping transduction modes of ANF-RGC, and for the first time demonstrate how differently they operate, and, yet generate cyclic GMP utilizing common CCD machinery.

## Introduction

Kick started by historic discovery of the prototype ANF-RGC (Atrial Natriuretic Factor Receptor Guanylate Cyclase) (Paul, 1986; Paul et al., 1987), the membrane guanylate cyclase (MGC) field has expanded to constitute a family of seven - -ANF-RGC, CNP-RGC, STa-RGC, ROS-GC1, ROS-GC2, ONE-GC and GC-G [reviewed (including) in terminology: (Sharma et al., 2016)]. By synthesizing cellular second messenger cyclic GMP the family is interlocked with the diverse physiological processes ranging from cardiac vasculature to cellular growth, sensory transductions, neural plasticity, temperature sensing and pineal-linked brain functions (reviewed and Figure 10 in Sharma et al., 2016). These processes are compromised by natural mutations incurred in their key genes (discussed in Duda et al., 2018); a few examples are: CNP-RGC-R^819^C causes acromesomelic dysplasia (Vasques et al., 2013; Nakao et al., 2015); and more than 100 ROS-GC1 mutations result in retinal dystrophies, mainly Leber’s congenital amaurosis (LCA1) and cone-rod degeneration (CORD6) (Perrault et al., 1996, 1999; Kelsell et al., 1998; Wilkie et al., 2000; Rozet et al., 2001; Hunt et al., 2010; Gradstein et al., 2016). A common molecular lesion of these abnormalities is loss of the basal catalytic activity to generate cyclic GMP (Duda et al., 1999a,b, 2000a; Wilkie et al., 2000).

The MGC signal transduction system is structurally and functionally different from the other two major signaling pathways, cyclic AMP and IP_3_, (Paul et al., 1987; Figure 2 in Sharma and Duda, 2014). Instead of three, it is a one component transduction system. A single transmembrane-spanning protein is crafted with a multi-modular design. This design allows it to be elastic, to exist in multiple forms, and to be multifunctional. MGC family consists of three subfamilies. (1) The original, Surface Receptor. It inherits a unique trait of being both a hormone receptor and a catalyst, guanylate cyclase. It contains three members - - ANF-RGC (Paul et al., 1987; Chinkers et al., 1989; Lowe et al., 1989; Pandey and Singh, 1990; Duda et al., 1991), the receptor for hormones ANF and BNP; CNP-RGC (Chang et al., 1989; Schulz et al., 1989; Duda et al., 1993), the receptor for hormone CNP; and STa-RGC (de Sauvage et al., 1991; Singh et al., 1991), the receptor for heat-stable enterotoxin (STa) and also for hormones guanylin and uroguanylin. (2) The [Ca^2+^]_i_-modulated ROS-GC, with two members, ROS-GC1 (Margulis et al., 1993; Duda et al., 1994) and ROS-GC2 (Lowe et al., 1995; Goraczniak et al., 1997; reviewed in Sharma and Duda, 2012). Its unique characteristic is that as of yet the function of their extracellular domains is unknown and their activities are under control of intracellular Ca^2+^ through specific calcium sensors. (3) The Ca^2+^-modulated and odorant receptor, with one member, ONE-GC (Duda et al., 2001, 2004; Hu et al., 2007; Leinders-Zufall et al., 2007; Duda and Sharma, 2008; Sharma and Duda, 2010). Its signature characteristic is that it is a quadric-modal transducer of: uroguanylin, [Ca^2+^]_i_-modulated myristoylated neurocalcin δ, GCAP1, and gaseous CO_2_. Notably, the gene encoding ONE-GC is rodent-specific, not present in the humans (Torrents et al., 2003; Caenepeel et al., 2004; Young et al., 2007).

Despite these distinctive features, the general topography of the subfamilies is similar. Each is composed of three domains: Extracellular (ExtD), Transmembrane (TMD) and Intracellular (ICD) (reviewed in Sharma et al., 2016). ICD is further subdivided into two vaguely defined domains, N-terminal KHD (Kinase Homology Domain) and C-terminal catalytic domain (CCD). Because KHD terminology was imprecise- - it has recently been redefined (discussed in Ravichandran et al., 2017; Duda et al., 2018). The 43-residue α-helical region located at its C-terminus is no longer considered to be a part of it but is now recognized as an individual module, termed the signaling helix domain (SHD) (Anantharaman et al., 2006). Its structure is conserved in MGC family, and it serves as a critical transmission switch to the catalytic domain for the downstream signaling pathways (Venkataraman et al., 2008; Saha et al., 2009; Zagel et al., 2013; Ravichandran et al., 2017; Duda et al., 2018). With in-depth definitions of the CCD structural and functional boundaries (Ravichandran et al., 2017), a new signal transduction paradigm of the MGC family members has been defined. It negates the old notion that trajectory of all signals from their origin to the catalytic domain is only downstream (reviewed in Sharma et al., 2016). And, demonstrates, that with exception of the surface receptor subfamily, the signal trajectory in other subfamilies is bi-directional, downstream and upstream (Ravichandran et al., 2017; Duda et al., 2018). This happens because the ROS-GC and ONE-GC subfamilies contain an extra C-Terminal Extension (CTE) domain beyond its catalytic domain. This domain wedges the catalytic domain between SHD and CTE, and CTE attains an independent status of being a modular domain. Consequently, three modes of signal transduction pathways can, and, do occur- -(1) downstream, (2) upstream, (3) mainstream or direct (the signals originate and are translated at the catalytic domain); the last situation in the case of ANF-RGC (Duda et al., 2012a), as explained below.

When discovered, ANF-RGC was declared to be uniquely regulated by the hormone ANF (Paul, 1986; Paul et al., 1987) and its function was linked with the physiological processes of diuresis and blood pressure regulation (de Bold, 2011). Implicit in this conclusion was that ANF-RGC machinery is solely structured to generate and transduce the extracellular hormonal signal. This conclusion, however, was recently revised. In this revelation, ANF-RGC also transduced an intracellularly generated Ca^2+^ signal; this signal is modulated through Ca^2+^-sensor myristoylated neurocalcin δ (myr-NCδ) and is physiologically linked with blood pressure regulation (Duda et al., 2012b). Aberration in this signaling pathway causes hypertension and cardiac myopathy in the mice (Duda et al., 2012b). Thus, ANF-RGC is a bimodal signal transducer, yet both modes have identical physiological outcome.

A unified structural theme of the MGC family is that it is homo-dimeric (reviewed in Sharma, 2010; Sharma et al., 2016). Biochemical details on MGC structure are limited, derived only from crystal structure of the extracellular domain of ANF-RGC (Ogawa et al., 2004) and from bimolecular fluorescence complementation (Duda et al., 2012c), modeling, and mutational analyses of the isolated catalytic module of ROS-GC1 (Liu et al., 1997; Venkataraman et al., 2008; Ravichandran et al., 2017). The crystal and single particle electron microscopy data demonstrate that the homo-dimeric structure of the extracellular domain is parallel (N-terminus to N-terminus and C-terminus to C-terminus) head-to-head (van den Akker et al., 2000; Ogawa et al., 2004; Ogawa et al., 2009). The bimolecular fluorescence complementation analysis shows that the monomers of the CCD form antiparallel dimers (Duda et al., 2012b) and the modeling analyses demonstrate that the two monomers assume a two-fold symmetry axis with a central gap of a wreath- or circlet-like shape (Liu et al., 1997; Rauch et al., 2008; Ravichandran et al., 2017). Notably, the plasma membrane anchoring is obligatory for the catalytic domain to manifest its full activity; without it, it is about one-order of magnitude lower (Ravichandran et al., 2017). Thus, the individual MGC modules do not show uniformity of existing in parallel or antiparallel orientation; and markedly, except for these two modules, status of the others is unknown.

Obligatory for ANF signaling of ANF-RGC catalytic activity is ATP (Chinkers et al., 1991; Marala et al., 1991). Mechanistically, ANF binding to the extracellular domain signals ATP binding to the intracellular ATP Regulated Domain (ARM), causing its allosteric modification and through a cascade of structural modifications involving ^669^WTAPELL^675^ motif activating the catalytic domain and accelerating the production of cyclic GMP (Goraczniak et al., 1992; Burczynska et al., 2007; Duda et al., 2009; reviewed in Sharma, 2010).

All signals, including the downstream, upstream and direct are translated at the common catalytic center consisting of 7 residues from each monomer of the MGC existing in the form of a wreath or circlet (Liu et al., 1997; Ravichandran et al., 2017). In resting state, the wreath is in an open conformation but in response to a regulatory signal it acquires a closed conformation optimal for accelerated formation of cyclic GMP from GTP.

The present study was designed to deepen our knowledge on the basic principles that make the hormonal and the Ca^2+^-dependent mechanisms of ANF-RGC activation distinct. The study establishes a critical role of SHD in maintaining the basal catalytic activity of ANF-RGC and in its hormonal but not Ca^2+^-dependent modulation.

## Materials and Methods

### Construction of Heptad Deletion and Site-Specific Mutants

The ANF-RGC deletion and single substitution mutants were created by PCR using the cDNA of the wild type (wt) rat ANF-RGC (Duda et al., 1991) as template and the following mutagenic primers: for deletion of the first heptad (ΔH1 mutant): FW: 5′-AACATCCTGGACAACCTGGCTAACAACCTGGAGGAA-3′; REV: 5′-TTCCTCCAGGTTGTTAGCCAGGGTTGTCCAGGATGTT-3′; for deletion of the second heptad (ΔH2 mutant), FW: 5′-TCAAGCATGGAGCAGTATGTAGAGGAGAGAACACAA-3′; REV: 5′-TTGTGTTCTCTCCTCTACATACTGCTCCCATGCTTGA-3′; for deletion of the third heptad (ΔH3 mutant), FW: 5′-AACAACCTGGAGGAACTGTATCTGGAGGAGAAGCGC-3′; REV: 5′-GCGCTTCTCCTCCATACAGTTCCTCCAGGTTGTT-3′; for deletion of the fourth heptad (ΔH4 mutant), FW: 5′-GAGGAGAGAACACAAGCTGCTGAGGCCTTGCTTTAC-3′; REV: 5′-GTAAAGCAAGGCCTCAGCAGCTTGTGTTCTCTCCTC-3′; for deletion of the fifth heptad (ΔH5 mutant), FW: 5′-CTGGAGGAGAAGCGCAAAATTCTGCCTCACTCCGTG-3′; REV: 5′-CACGGAGTGAGGCAGAATTTGCGCTTCTCCTCCAG-3′; and for substituting the R^802^ residue with C (R^802^C mutant), FW: 5′-GAACTGGTAGAGGAGTGTACACAAGCTTATCTG-3′; REV: 5′-CAGATAAGCTTGTGTACACTCCTCTACCAGTTC-3′.

To delete the entire SHD, amino acid residues L^785^-Q^819^, from ANF-RGC (ΔSHD mutant) two *Hpa*I restriction sites were introduced in ANF-RGC cDNA; the first site was between codons for L^784^ and L^785^, the second between Q^819^ and I^820^. The mutated ANF-RGC cDNA was digested with *Hpa*I and re-ligated.

All mutations were verified by sequencing.

### Expression in a Heterologous Cell System

COS-7 cells were induced to express ANF-RGC or its mutants using Lipofectamine (Thermo Fisher Scientific). Seventy hours after transfection, the cells were harvested and their membranes prepared (Duda et al., 2018).

### ANF-RGC Guanylate Cyclase Catalytic Activity Assays

Membrane samples of transfected COS cells were incubated individually without or with 10^-7^ M ANF and varying concentrations of ATP or varying concentrations of recombinant myristoylated neurocalcin d (myrNCδ). MyrNCδ was purified as described in Duda et al. (2012b). The assay mixture (25 ml) consisted of (mM): 10 theophylline, 15 phosphocreatine, and 50 Tris-HCl; pH 7.5, and 20 μg creatine kinase (Sigma). In experiments with myr-NCδ, 1 μM Ca^2+^ was present in the reaction mixture. The reaction was initiated by addition of the substrate solution 4 μM MgCl_2_ and 1 μM GTP (final concentrations) and maintained by incubation at 37°C for 10 min. In the experiments for [GTP] dependency of guanylate cyclase activity, the GTP concentrations varied from 0 to 2 μM and the MgCl_2_ concentration was constant, 4 μM. The reaction was terminated by the addition of 225 ml of 50 μM sodium acetate buffer, pH 6.2, followed by heating on a boiling water bath for 3 min. The amount of cyclic GMP formed was determined by radioimmunoassay (Nambi et al., 1982). All assays were done in triplicate and except where stated otherwise, were performed three times. Guanylate cyclase activity is presented as average ± SD of these experiments. The EC_50_ values were determined from the experimental values by GraphPad PRISM program. The effect of different conditions on ANF-RGC activity was evaluated by performing a one-way ANOVA; *p*-values ≤ 0.05 were considered to be significant.

To correlate the catalytic changes brought about by the mutations, the activities of the mutants were compared with wild type recombinant ANF-RGC through Michaelis plots for the ligand used by fitting the data with the Hill equation, v = V_max__X_ (S)^n^/ (K_M_)^n^ + (S)^n^. V_max_ is the maximal activity, S is the concentration of the ligand, K_M_ is the ligand concentration at which half-maximal velocity is achieved, and n is the Hill coefficient.

### Western Blot

After boiling in a gel-loading buffer (62.5 μM Tris-HCl, pH 7.5, 2% SDS, 5% glycerol, 1 μM β-mercaptoethanol, and 0.005% bromophenol blue) 50 μg of membrane protein was subjected to SDS-polyacrylamide gel electrophoresis in a buffer (pH 8.3) containing 0.025 M Tris, 0.192 M glycine, and 0.1% SDS. The proteins were transferred to immobilon membranes (Millipore) in the same buffer but containing 5% methanol. The blot was incubated in Tris-buffered saline (TBS, pH 7.5) containing 100 μM Tris-HCl, 0.9% NaCl, and 0.05% Tween-20 (TBS-T) with 5% bovine serum albumin (blocking buffer) overnight at 4°C and rinsed with TBS-T. The anti-ANF-RGC antibody (raised in rabbit; Santa Cruz Biotechnology, Inc.) was added at 1: 2000 dilution in the blocking buffer and the incubation was continued overnight at 4°C. After the blot was rinsed with TBS-T, the incubation was continued with peroxidase-conjugated AffiniPure goat anti-rabbit IgG (Jackson ImmunoResearch Laboratories, Inc.) diluted 1: 50,000 for 1 h at room temperature. Finally, the blot was treated with SuperSignal West Dura Extended Duration Substrate (Thermo Scientific) for 5 min according to the manufacturer’s protocol. The immunoreactive band was detected by exposing the blot to CL-XPosure film (Thermo Scientific).

### ANF-RGC Catalytic Efficiency

The protocol is described in Duda et al. (2012b). Briefly, aliquots of 10 to 0.1 ng of the antigen for the ANF-RGC antibody (ANF-RGC fragment aa 486–661) were diluted in Laemmli sodium dodecyl sulfate sample buffer and loaded next to 50 μg (total protein) of COS cell membranes expressing ANF-RGC. After electrophoresis the proteins were transferred to immobilon membranes (Millipore) and immunostained with anti-ANF-RGC antibody as described above and exposed to X-ray film. The amount of ANF-RGC in COS cell membranes was quantified from the calibration curve produced by the antigen standards. COS cell membranes from the same transfection experiment were assayed for guanylate cyclase activity.

The catalytic efficiencies of the mutants were calculated by comparing their expression levels in COS cells and basal catalytic activities with wt-ANF-RGC.

### Molecular Modeling

ANF-RGC (Gene: Npr1) is a homo-dimer protein, the amino acid sequence segment N^783^- P^822^ was analyzed for the features characteristic of SHD domain. Note that the amino acid numbering shown above corresponds to the mature ANF-RGC protein (Duda et al., 1991) which is identical to the UniProt P18910-1 full-length native sequence segment, 811–850. Due to lack of experimental structure for SHD domain, three-dimensional structural models were built using I-TASSER (Iterative Threading and ASSEmbly Refinement; web server version)^1^ software. I-TASSER is an automated protein 3D structure prediction program. It employs a hierarchical protein modeling approach that uses secondary structural data, template identification, profile-profile threading and iterative threading assembly refinement steps to build 3D protein models. I-TASSER identified five unique 3D-structural templates (PDB IDs), 1zxaA, 3hlsA, 4pkyB, 5svaV, and 1y11 for the SHD domain. Structure summary of the templates indicated that they all belong to a coiled-coiled domain family. Based on the secondary structure predictions (Supplementary Figure S1) and the I-TASSER C-score, the top-ranked monomer was selected as the representative structure and hereafter referred to as the default model for SHD sequence.

The dimer models for the SHD signaling helix were built and analyzed using protein-protein docking programs, M-ZDOCK (Radially Symmetric Multimer Docking software)^2^ and Z-DOCK^3^. The SOCKET software^4^ was used to search for the most energy favorable packing structures from the top ten M-ZDOCK dimer models (the details are provided in the Supplementary Figures S2, S3).

## Results

### Structure-Focused View of the SHD

**FIGURE 1:**
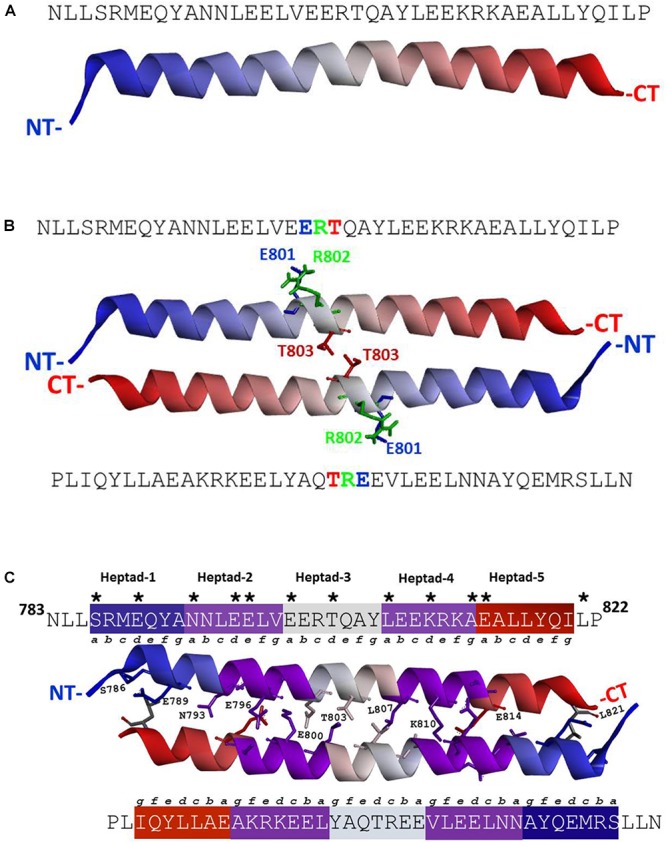
**(A)** ANF-RGC region amino acid residues N^783^-P^822^ exists as a α-helix. Three-dimensional structural model for ANF-RGC region comprising amino acid residues N^783^-P^822^ was built using I-TASSER (https://zhanglab.ccmb.med.umich.edu/I-TASSER/) protein modeling software. The top-ranked model is shown. The protein is displayed in a solid ribbon style with a continuous gradient coloring scheme that starts with dark blue at the N-terminus (NT) progressing to white and ends as dark red at the C-terminus (CT). **(B)** ANF-RGC region amino acid residues N^783^-P^822^ forms an anti-parallel dimer. The dimer model for the ANF-RGC region amino acid residues N^783^-P^822^ was built using a protein-protein docking program, M-ZDOCK (Symmetric Multimer Docking software; http://zdock.umassmed.edu/m-zdock). Top-ranked M-ZDOCK predicted complex is shown. The E^801^R^802^T^803^ – triad that is important for the dimer interface is shown in stick form and marked with green, blue and red colors. The protein chains coloring style is same as the figure. **(C)** ANF-RGC region amino acid residues N^783^-P^822^ forms an anti-parallel coiled coil. Fourth ranked M-ZDOCK complex was identified by SOCKET (http://coiledcoils.chm.bris.ac.uk/socket/) as the best dimer model that satisfies the Knobs-into-Holes mode of packing between alpha-helices – a unique feature of coiled-coils. Protein chains are displayed in solid ribbon. The interface residues, 3 Å from each chain, are identified with asterisk in the linear sequence. They are the same for both chains and are shown in stick form and labeled except for two residues, E^797^ and A^813^, shown in stick-mode but are not labeled for clarity of the figure. For the same reason, only the interface residues from the top chain are labeled.

According to the previously defined characteristics of the individual residues (Anantharaman et al., 2006), the ANF-RGC segment, N^783^- P^822^ [mature protein numbering (Duda et al., 1991)], meets the criteria of being the SHD. To verify this, in absence of any crystal structure information, a three-dimensional model of the N^783^- P^822^ region was built employing a hierarchical approach of structural template identification, threading and iterative template fragment assembly simulations. The secondary structure prediction (PSSpred)^5^ and I-TASSER models demonstrate that this segment exists as a single α-helix (Figure 1A and Supplementary Figure S1).

Since ANF-RGC, like other membrane guanylate cyclases, exists as a homodimer with its catalytic domain in anti-parallel, wreath-like orientation (Ravichandran et al., 2017), the question was: what is the configurational positioning of the monomers within the SHD dimer? Two molecular docking programs (M-ZDOCK^2^ and Z-DOCK^3^) were used for the analyses. M-ZDOCK (a symmetric dimer prediction software) (top-10) predicted an antiparallel arrangement (Figure 1B and Supplementary Figure S3). Up to 30 top ranked M-ZDOCK predicted conformations were analyzed and all of them showed anti-parallel orientations. We also repeated the modeling using a related docking software, Z-DOCK. Unlike with M-ZDOCK, we found some parallel docked conformations (4 out of top-10 ranked complexes). Altogether, based on the modeling results and the topology of the ANF-RGC intracellular domains (TM, ARM and SHD), we believe that antiparallel orientation could be a possible packing form for SHD. Notably, the conserved signature ^801^ERT^803^ motif (shown in Figure 1B in stick form and marked with blue, green, and red colors for the E, R, and T residues, respectively) is centrally located what constitutes to be one of the distinctive features of the SHD (Anantharaman et al., 2006). The antiparallel homodimers were then analyzed for the existence of heptad arrangement because it has been proposed to be critical for the SHD coiled-coil structure and function (Anantharaman et al., 2006). The SOCKET software (Walshaw and Woolfson, 2001) was utilized to search for the most energy favorable packing structures between the two alpha-helices. Out of the top ten M-ZDOCK predicted complexes (Supplementary Figure S3), one (4th rank) was identified to satisfy the three-dimensional arrangement of the heptad residues in a coiled-coil, for example: i and i+7 residues face the same direction and the coiled coil dimer interface is created using interactions between “a” and “d” heptad residues. The other complexes (shown in the Supplementary Figure S3) did not satisfy this arrangement. Detailed SOCKET analysis of top-ranked M-ZDOCK structure that satisfies the coiled coil helix conditions is provided in the Supplementary Figure S4. Thus, this homo-dimer structure comprising five repeating heptads was chosen as the best possible model for the analyzed region. The residues that could most likely (at 3 Å cut-off) form the dimer interface were: S^786^, E^789^, N^793^, E^796^, E^797^, E^800^, T^803^, L^807^, K^810^, A^813^, and E^814^. These residues and their locations within the respective heptads are shown in the three-dimensional homo-dimer model (Figure 1C) and their possible interactions are listed in Table 1.

**Table 1:** Key interactions between amino acid residues forming the interface of ANF-RGC SHD as identified by M-ZDOCK program.

**ChainA**	**ChainB**
GLU796	LYS810, ALA813, GLU814, LEU817
GLU800	LEU807, LYS810, ARG811, GLU814
LYS810	GLU796, VAL799, GLU800, THR803
GLU814	ASN793, GLU796, GLU797, GLU800
**ChainB**	**ChainA**
GLU796	LYS810, ALA813, GLU814, LEU817
GLU800	LEU807, LYS810, ARG811, GLU814
LYS810	GLU796, VAL799, GLU800, THR803
GLU814	ASN793, GLU796, GLU797, GLU800


Since the modeling studies demonstrated that the ANF-RGC sequence segment N^783^-P^822^ satisfy all structural requirements of being a SHD, the next logical step was to scrutinize its functional significance in ANF-RGC signal transduction. Through sequential deletion and expression analyses, the roles of the entire SHD and each individual heptad in modulation of the ANF/ATP-dependent and the Ca^2+^-dependent signaling pathways were investigated.

### Heptad-Deletion Analysis on the Basal Catalytic Activity of CCD

**FIGURE 2:**
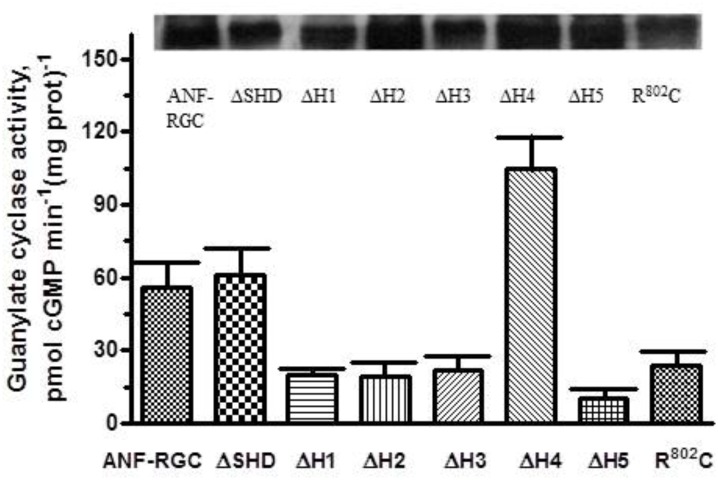
Deletion of individual heptads but not of the entire SHD of the ANF-RGC affects the cyclase basal catalytic activity. ANF-RGC deletion mutants were prepared as described in Section “Materials and Methods.” Wt-ANF-RGC and the mutants were individually expressed in COS cells which membranes were assayed for guanylate cyclase activity. The experiment was done in triplicate and repeated three times. The results presented are average ± SD of these experiments. The expression of the proteins was monitored by Western blot. The intensities of the ANF-RGC immunoreactive bands of the expressed proteins are shown at the top of the figure.

Six deletion mutants - -ΔSHD, ΔH (Heptad)1, ΔH2, ΔH3, ΔH4, ΔH5- - were constructed. The wt-ANF-RGC and the deletion mutants were expressed in COS cells and their BASAL catalytic activities were determined in the cells particulate fractions (Figure 2). Each activity is denoted as [pmol cGMP min^-1^ (mg prot)^-1^]. The basal catalytic activity of wt-ANF-RGC was 56 ± 9 pmol cGMP min^-1^ (mg prot)^-1^, K_M_ for the substrate GTP, 0.63 μM and Hill coefficient 2.1 (Table 2). These values served as a control. Notably, divergence in a mutant’s basal catalytic characteristics from the control values will measure the deviation from the optimal positioning of one or more of the seven catalytic elements formed at the core catalytic domain homodimer interface: D^849^ and D^893^ (Mg^2+^ binding), N^968^ (ribose positioning), E^879^ and C^951^ (guanine recognition), and/or R^940^ and R^972^ (triphosphate positioning) (Ravichandran et al., 2017).

**Table 2:** Deletion of the individual heptads or the entire SHD from ANF-RGC signaling helix does not affect of the cyclases’ catalytic characteristics.

	K_M_ [mM]	Hill’s coefficient	K_cat_
wt-ANF-RGC	0.63 ± 0.12	2.1 ± 0.3	6.5 ± 0.3
ΔSHD	0.59 ± 0.11	2.1 ± 0.3	7.1 ± 0.5
ΔH1	0.65 ± 0.13	2.1 ± 0.2	2.3 ± 0.2
ΔH2	0.60 ± 0.05	1.8 ± 0.1	2.3 ± 0.2
ΔH3	0.70 ± 0.14	2.1 ± 0.3	2.6 ± 0.3
ΔH4	0.66 ± 0.09	2.2 ± 0.2	12.2 ± 1.1
ΔH5	0.62 ± 0.10	1.7 ± 0.2	1.2 ± 0.1
R 802 C	0.61 ± 0.13	1.9 ± 0.3	2.8 ± 0.3


*COS cells were induced to express ANF-RGC or its mutants. Membranes of these cells were individually analyzed for guanylate cyclase activity in the presence of increasing concentrations of GTP (0–2 μM) and constant 4 μM MgCl_2_. The experiment was done in triplicate and repeated four times. Presented values of K_*M*_ ± SD were determined by GraphPad PRISM 4 program, of the Hill’s coefficients ± SD and K_*cat*_ ± SD were calculated as described in Section “Materials and Methods.”*

#### ΔSHD

The mutant exhibited basal catalytic activity of 61 ± 10 pmol cGMP min^-1^ (mg prot)^-1^ (Figure 2). Its activity was dependent on GTP concentration with a K_M_ for GTP equal to 0.6 μM and the Hill coefficient 2.1 ± 0.3. These values are almost identical to the control ones. In conclusion, the whole SHD structure has no role in controlling the basal catalytic activity of CCD.

#### ΔH1, ΔH2, ΔH3

Deletion of these heptads resulted in lowering of the basal activities to 20 ± 1, 19 ± 4, and 22 ± 5 pmol cGMP min^-1^ (mg prot)^-1^ (Figure 2). These respective values demonstrate that in the deletion mutants, the alignment of the 7-residue catalytic element in the CCD homodimer structure has been disturbed and suggest that these three heptads in their native states cause about three-fold upregulation of the basal catalytic activity.

#### ΔH5

Deletion of the 5th heptad caused lowering of the basal activity to 10 ± 3 pmol cGMP min^-1^ (mg prot)^-1^ (Figure 2). This value indicates that the 5th heptad controls more than 80% of the basal catalytic activity of CCD, suggesting that its deletion severely compromises alignment of the 7-residue catalytic element.

#### ΔH4

Deletion of the 4th heptad resulted in basal catalytic activity of 105 ± 18 pmol cGMP min^-1^ (mg prot)^-1^ (Figure 2). This activity, significantly higher than that of the wt-ANF-RGC indicates that this heptad, in contrast to the other four, functions as a suppressor of ANF-RGC activity. It prevents the 7-residue catalytic element from acquiring the optimal for catalysis conformation but does not interfere with the substrate GTP binding as the K_M_ for GTP remains comparable to that of wt-ANF-RGC.

To verify that these differences in basal catalytic activities truly reflect the differences of enzymatic properties but not different levels of the proteins expression, the membranes were analyzed by Western blot (Figure 2: the intensities of the immunoreactive bands are shown at the top of the figure).

When the heptad-deletion mutants’ catalytic velocities were analyzed, they showed identical relationships between their catalytic activities and the substrate, GTP, concentration. The GTP concentration at which the activities of the wt-ANF-RGC and the deletion mutants were equal one-half of the maximal activity (K_M_) was ∼0.65 μM and the Hill coefficient values were similar, averaging at ∼2 (Table 2).

We therefore, conclude that in ANF-RGC the SHD tunes the basal catalytic activity of the CCD module. In basal (unstimulated) state, SHD tightly regulates configuration of the wreath- or circlet-like structure of the CCD formed by the 7-residue catalytic element at the interface of its homo-dimer. The tuning is critical, because its individual heptad regions in isolation cause disharmony in functioning of the CCD and generation of cyclic GMP in its resting state.

### ^801^E^802^R^803^T in H3 Is a Critical Conserved Motif in the MGC Family

The original signature characteristic defined for the SHD was that its third heptad contained a strongly conserved “ERT” motif (Figure 1B). The motif is also present in all MGC family members. In ROS-GC1 the third heptad is the “hot-spot” for naturally occurring mutations which ultimately lead to blindness with the R^787^ being the most mutation-prone residue as four single mutations and two complex mutations are known to affect it (reviewed in Sharon et al., 2017). R^787^C mutation disrupts H3’s structural integrity, disables the basal ROS-GC1’s catalytic activity and causes a cone-rod degeneration (Kelsell et al., 1998; Duda et al., 2000a).

In ANF-RGC the R^802^ residue corresponds to R^787^ in ROS-GC1. No naturally occurring mutation at this position as yet has been recorded in ANF-RGC. However, to test if the concept on the critical significance of the R residue in the function of MGC is broadly applicable, ANF-RGC R^802^C mutant was constructed and analyzed.

To assure that the introduced mutation did not impair proper membrane targeting, the wt-ANF-RGC and the mutant were expressed in COS cells and analyzed by Western blots using anti-ANF-RGC antibody (Figure 2). In both cases, immunoreactivity was found in the plasma membranes and in almost quantitatively equivalent amounts.

In contrast to its parent wt-ANF-RGC’s basic catalytic activity of 56 pmol cyclic GMP min^-1^ (mg prot)^-1^, the mutant’s activity was only 24 pmol cyclic GMP min^-1^ (mg prot)^-1^, the value almost identical to that for ΔH3 mutant (Figure 2). The K_M_ for GTP and Hill coefficient values were 0.61 ± 0.13 μM and 1.9 ± 0.3, respectively, very close to those for the wt-ANF-RGC (Table 2). These results demonstrate that like ΔH3, R^802^C mutation lowers the basal catalytic activity of ANF-RGC and concomitantly destroys the structural integrity of H3 and SHD. At the sub-molecular level, R^802^ residue in H3 appears to be the real determinant of the modulation of CCD’s 7-residue catalytic element. This interpretation is supported by the finding where the analogous mutation in ROS-GC1 changes its structural configuration from the active homodimer to the inactive monomer form (Duda et al., 2000a).

### ANF-RGC Regulatory Activity

Signaling helix domain was predicted to always exist between two signaling domains where it separated the N-terminal sensory domain from the C-terminal catalytic signaling domain (Anantharaman et al., 2006). This theoretical prediction was supported experimentally with the ROS-GC1 transduction system (Duda et al., 2012b). Deletion of SHD blocked the downstream flowing (N-terminal-origin) GCAP1-modulated Ca^2+^ signal, yet not the upstream flowing GCAP2-modulated Ca^2+^ signal that did not pass through the SHD. Here we test whether this prediction is also true for two ANF-RGC regulatory systems, ANF-modulated and Ca^2+^-modulated.

#### ANF/ATP-Dependent ANF-RGC Regulatory Activity

Binding of ANF to the extracellular receptor domain of ANF-RGC triggers a chain of structural changes which are carried through the transmembrane domain to the intracellular portion of the cyclase where, by ATP-dependent changes within the ARM domain, they are processed further, sensed by the SHD and finally translated into the production of cyclic GMP at the catalytic domain.

##### Intact SHD structure is obligatory for signaling

**FIGURE 3:**
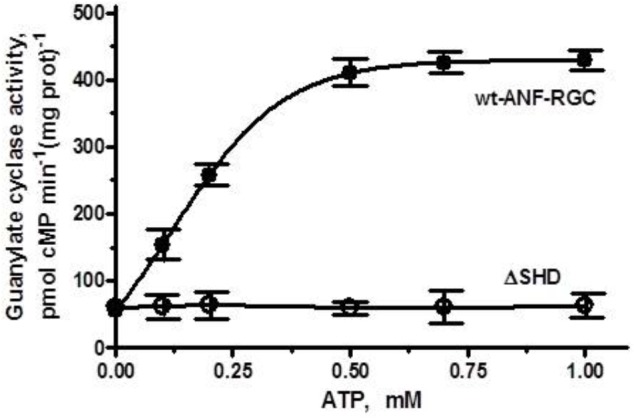
The SHD is obligatory for ANF/ATP signaling of the ANF-RGC catalytic activity. COS cells were induced to express the ΔSHD mutant and their membranes were assayed for guanylate cyclase activity in the presence of 10^-7^ M ANF and indicated concentrations of ATP. Membranes of COS cells expressing wt-ANF-RGC were processed identically as control. The experiment was done in triplicate and repeated three times. The results presented are average ± SD of these experiments. The error bars are indicated.

When the membranes of COS cells expressing ΔSHD mutant were exposed to 10^-7^ M ANF and increasing concentrations of ATP, the activity of the mutant did not increase but stayed constant at ∼ 61–63 pmol cyclic GMP min^-1^ (mg prot)^-1^ within the 0–1 μM range of ATP concentrations tested (Figure 3). Under identical conditions, membranes of COS cells expressing wt-ANF-RGC showed 10^-7^ M ANF and ATP dose-dependent increase of guanylate cyclase catalytic activity (Figure 3). Since deletion of the total SHD renders the cyclase completely unresponsive to ANF/ATP it is concluded that the intact SHD is obligatory for the transduction of the signal originating at the cyclase’s extracellular domain.

##### Each heptad differentially impacts signaling

Wt-ANF-RGC and the deletion mutants were expressed in COS cells and challenged with 10^-7^ M ANF and varying concentrations of ATP. The results were analyzed in terms of the maximal catalytic activity achieved and the x-fold stimulation exhibited by the cyclases in response to the ligands. The EC_50_ values for ATP are provided in Table 3.

**Table 3:** The EC_50_ (±SD) values for ATP in the presence of 10^-7^M ANF and for myr-NCδ in the presence of 1 μM Ca^2+^, in modulating the catalytic activity of wt-ANF-RGC and its mutants.

	EC_50_	
	
	ATP (mM)	myr-NC8 *(∖iM)*
wt-ANF-RGC	0.25 ± 0.01	0.5 ± 0.03
ΔSHD	none	0.4 ± 0.01
ΔH1	0.25 ± 0.02	0.7 ± 0.04
ΔH2	0.28 ± 0.01	0.9 ± 0.03
ΔH3	0.25 ± 0.01	0.8 ± 0.02
ΔH4	0.25 ± 0.02	0.5 ± 0.02
ΔH5	0.25 ± 0.02	0.7 ± 0.03
R ^802^ C	0.25 ± 0.01	0.7 ± 0.02


*The experiments were performed as described in Section “Materials and Methods” as well as in the respective figure legends.*

**FIGURE 4:**
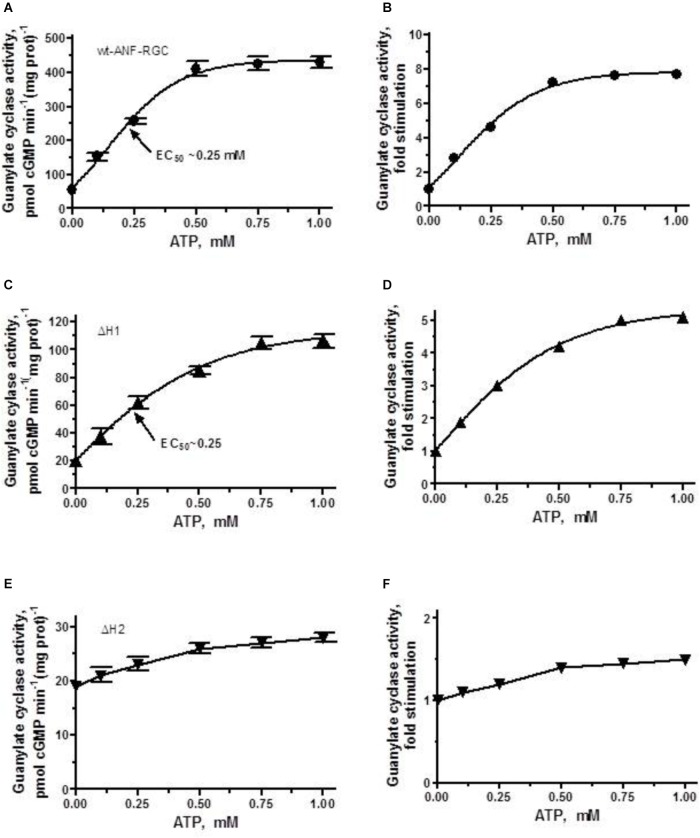
ANF/ATP effect on the catalytic activity of wt-ANF-RGC and its ΔH1 or ΔH2 mutants. COS cells were individually transfected with ANF-RGC, ΔH1 or ΔH2 mutants’ cDNAs and their membrane fractions were assessed for guanylate cyclase activity in the presence of 10^-7^ M ANF and increasing concentrations of ATP. **(A,C,E)** Show the specific guanylate cyclase activities of ANF-RGC, ΔH1 and ΔH2 mutants respectively, while figures **(B,D,F)**, their x-fold stimulations. The experiment was done in triplicate and repeated four times. The results shown are mean ± SD of these experiments. The error bars for **(A,C,E)** are shown whereas for **(B,D,F)** they are within the size of the symbols.

As expected, the activity of wt-ANF-RGC was stimulated in an ATP-dose-dependent fashion with half-maximal stimulation (EC_50_) at ∼0.25 μM ATP (Figure 4A). The maximal stimulation achieved was about 420 pmol cyclic GMP min^-1^ (mg prot)^-1^, what represents ∼7.5-fold increase above the basal level (Figure 4B).

The ΔH1 mutant responded to the ANF/ATP stimulation by dose-dependent increase in activity, with an EC_50_ at 0.25 μM ATP (Figure 4C). The maximal activity reached was ∼100 pmol cyclic GMP min^-1^(mg prot)^-1^, what with the mutant’s basal activity of 20 pmol cyclic GMP min^-1^ (mg prot)^-1^, corresponded to 5-fold increase (Figure 4D).

The ΔH2 mutant responded to ANF/ATP with only marginally increased catalytic activity. The increase was from 19 to 27 pmol cyclic GMP min^-1^(mg prot)^-1^ (Figure 4E) what amounted to no more then 1.4-fold stimulation (Figure 4F). These values demonstrate critical role of this heptad in both basal and ANF/ATP-regulated ANF-RGC activity.

**FIGURE 5:**
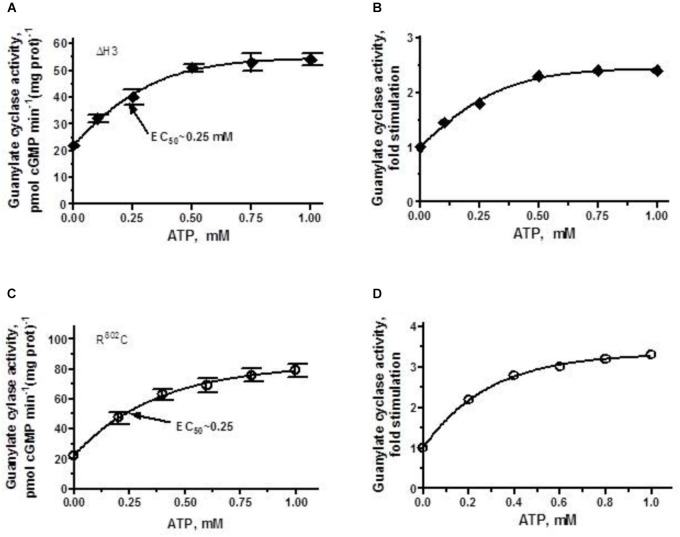
ANF/ATP effect on the catalytic activity of ANF-RGC ΔH3 and R^802^C mutants. Membranes of COS cells individually expressing ΔH3 or R^802^C mutants were assayed for guanylate cyclase activity in the presence of 10^-7^ M ANF and increasing concentrations of ATP. **(A,C)** Show respectively, the specific guanylate cyclase activities of the ΔH3 and R^802^C mutants, while figures **(B,D)**, their x-fold stimulations. The experiment was done in triplicate and repeated three times. The results shown are mean ± SD of these experiments. The error bars for **(A,C)** are shown whereas for **(B,D)** they are within the size of the symbols.

The ΔH3 mutant in addition to losing 60% of the wild-type cyclase’s activity (Figure 2) lost also most of its ability to be stimulated by ANF/ATP (Figure 5A). The stimulation of approximately 2.3-fold above the basal level (Figure 5B) reached plateau at ∼0.75 μM ATP with the V_max_ averaging at ∼50 pmol cGMP min^-1^ (mg prot)^-1^ and the half-maximal stimulation occurred at ATP concentration of 0.25 μM.

When the R^802^C mutant was exposed to 10^-7^ M ANF and increasing concentrations of ATP, its activity rose in a dose-dependent fashion (Figure 5C), but the stimulation was limited, not exceeding 3-fold (Figure 5D). Thus, deletion of the third heptad or R^802^C mutation, similar to R^787^C mutation in ROS-GC1, significantly disables the ANF-RGC’s basic catalytic activity [lowers it from 56 pmol cyclic GMP min^-1^ (mg prot)^-1^ for the wt-ANF-RC to 22 and 24 pmol cyclic GMP min^-1^ (mg prot)^-1^ for the ΔH3 and R^802^C mutants, respectively]. The picture is different however, when analyzing the ligand-dependent activity. The R^802^C mutation in ANF-RGC disables the cyclase’s responsiveness to ANF/ATP thus, makes it hyporesponsive to the physiological ligand, whereas in ROS-GC1 the R^787^C mutation makes the cyclase hyper-responsive to its physiological ligand GCAP1 (Duda et al., 2000a, 2018).

**FIGURE 6:**
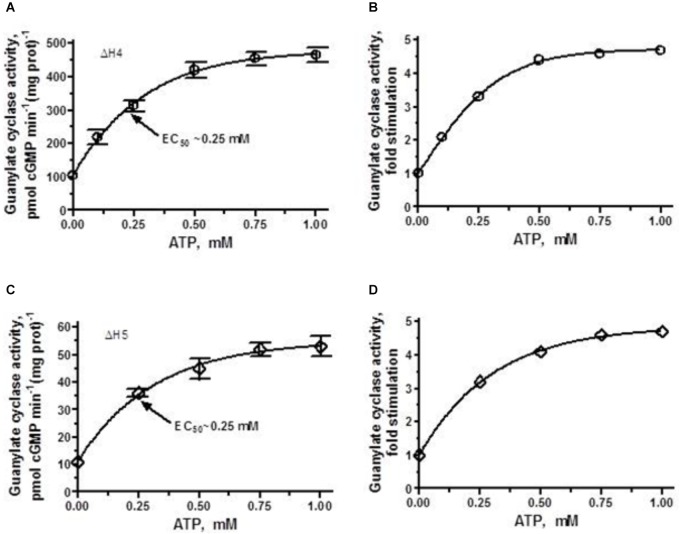
ANF/ATP effect on the catalytic activity of ΔH4 and ΔH5 mutants. COS cells were individually transfected with ΔH4 or ΔH5 mutants and their membrane fractions were assayed for guanylate cyclase activity in the presence of 10^-7^ M ANF and increasing concentrations of ATP. **(A,C)** Show the specific guanylate cyclase activities of the ΔH4 and ΔH5 mutants, respectively while figures **(B,D)**, their x-fold stimulations. The experiment was done in triplicate and repeated three times. The results shown are mean ± SD of these experiments. The error bars for **(A,C)** are shown whereas for **(B,D)** they are within the size of the symbols.

ΔH4 mutant, which basal activity exceeds that of the parental ANF-RGC, in the presence of 10^-7^ M ANF and 0–1 μM ATP was further stimulated, in a dose-dependent fashion, in its catalytic activity and ultimately reaches the point of saturated activity of ANF-RGC (compare Figures 4A, 6A). Despite achieving the same V_max_ as the wt-ANF-RGC, due to its elevated basal activity, the ΔH4 mutant was stimulated only 4.5-fold above the basal activity (Figure 6B).

ΔH5 mutant, characterized by the lowest of all heptad-deletion mutants basal activity of 10 pmol cyclic GMP min^-1^ (mg prot)^-1^ was responsive to ANF/ATP. When challenged with 10^-7^ M ANF and increasing concentrations of ATP its activity rose in a dose-dependent fashion (Figure 6C). Although the x-fold stimulation was significant, 5-fold (Figure 6D) the V_max_ achieved amounted to no more than the basal activity of the wt-ANF-RGC.

#### Ca^2+^/Neurocalcin δ Dependent ANF-RGC Regulatory Activity

**FIGURE 7:**
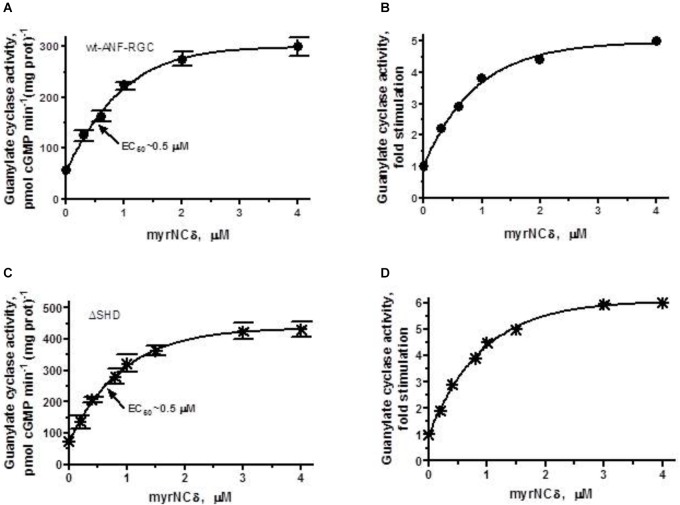
The SHD has no role in Ca^2+^/myr-NCδ signaling of the ANF-RGC catalytic activity. COS cells were individually induced to express wt-ANF-RGC or its ΔSHD mutant. Membranes of transfected cells were exposed to increasing concentrations of myr-NCδ in the presence of 1 μM Ca^2+^ and assayed for the guanylate cyclase activity. **(A,C)** Show the specific guanylate cyclase activities of ANF-RGC and its ΔSHD mutant, respectively while figures **(B,D)**, their x-fold stimulations. The experiment was done in triplicate and repeated three times. The results shown are mean ± SD of these experiments. The error bars for **(A,C)** are shown whereas for **(B,D)** they are within the size of the symbols.

Given that SHD is the gateway module for the downstream hormonal (ANF/ATP) signaling pathway, does it have any role in the mainstream Ca^2+^/myristoylated neurocalcin δ (myr-NCδ)-modulated signaling pathway? This question is critical because these two pathways originate in the modules at the opposite ends of the SHD, the hormonal in the extracellular domain and the Ca^2+^ in CCD [Figure 11 in Duda et al., 2012a; Figure 7; in Review: (Sharma et al., 2016)].

To answer this question, first, the recombinant wt-ANF-RGC (control) and ΔSHD mutant expressed in COS cells were individually reconstituted with myr-NCδ and the guanylate cyclase activity was measured. The reaction mixtures contained 1 μM of Ca^2+^ because in the absence of Ca^2+^, myr-NCδ is ineffective in modulating ANF-RGC activity (Duda et al., 2012b).

In accordance with previous observations (Duda et al., 2012a,b), Ca^2+^-bound myr-NCδ stimulated the catalytic activity of the wt-ANF-RGC in a dose-dependent fashion from 56 to 278 pmol cGMP min^-1^ (mg prot)^-1^ (Figure 7A); this equaled to 5-fold amplification of the basal activity (Figure 7B). The half maximal stimulation was achieved at 0.5 μM myr-NCδ (EC_50_) and the calculated Hill coefficient was 1.2. These values are very similar to those obtained previously (Duda et al., 2012b) and served as control for the analyses of ANF-RGC mutants’ responses. Because ANF-RGC like other membrane guanylate cyclases exists as a homodimer and only dimer of Ca^2+^-bound myr-NCδ activates it (Duda et al., 2012b), the Hill coefficient of 1.2 demonstrates that 1 dimer of Ca^2+^-bound myr-NCδ binds and activates 1 dimer of ANF-RGC.

ΔSHD mutant, in response to Ca^2+^-bound myr-NCδ behaved almost identically to the control wt- ANF-RGC. The mutant was stimulated in its catalytic activity up to 348 pmol cGMP min^-1^ (mg prot)^-1^ with a half maximal stimulation at 0.4 μM myr-NCδ (Figure 7C). The x-fold stimulation was 5.7 (Figure 7D) and the Hill coefficient for myr-NCδ, 1.08 ± 0.1. These results demonstrate that the SHD has no role in Ca^2+^-modulated myr-NCδ signaling of CCD.

**FIGURE 8:**
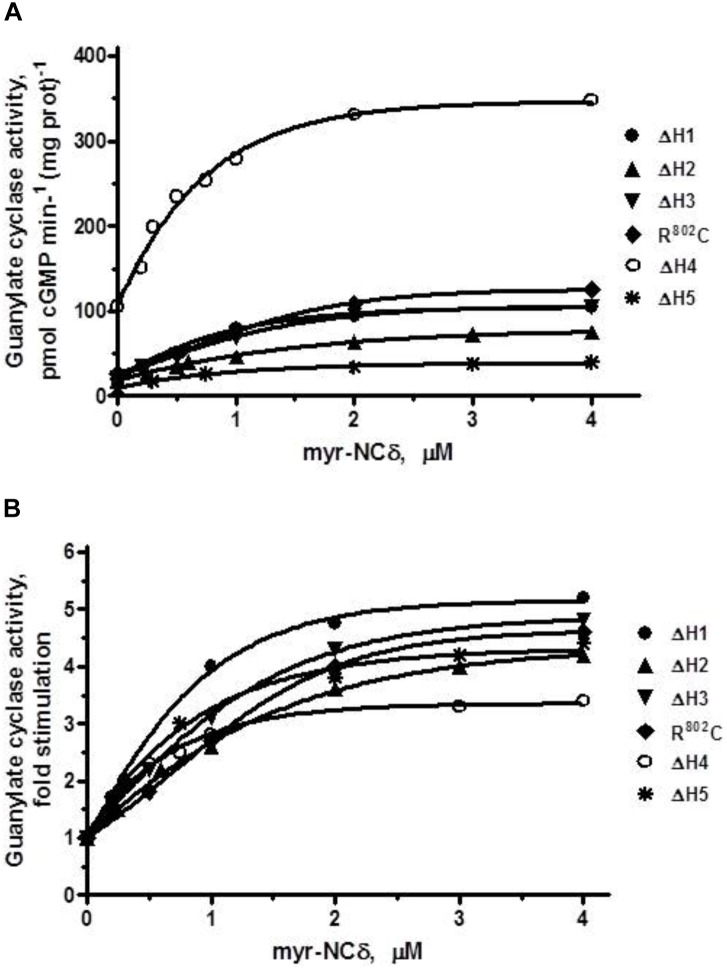
The effect of myr-NCδ on ANF-RGC heptad deletion (ΔH1 – ΔH5) and R^802^C mutants. COS cells were induced to individually express each of the ANF-RGC heptad deletion mutants: ΔH1, ΔH2, ΔH3, ΔH4, ΔH5, or the substitution R^802^C mutant. Membranes of transfected cells were exposed to increasing concentrations of myr-NCδ in the presence of 1 μM Ca^2+^ and assayed for the guanylate cyclase activity. **(A)** Shows the specific guanylate cyclase activities of the mutants whereas figure **(B)**, their x-fold stimulations. The experiment was done in triplicate and repeated three times. The results shown are mean ± SD of these experiments. The error bars are within the size of the symbols.

This being the case, it was predicted that individual building blocks of SHD- -H1, H2, H3, H4, H5- -will also have no regulatory role in Ca^2+^-modulated signaling, meaning that deletion of any individual heptad will not affect the x-fold stimulation which will remain approximately the same for all deletion mutants. To test this prediction the individual heptad deletion mutants were analyzed for their Ca^2+^-bound myr-NCδ-dependent activity. Because the results were very similar (Figure 8) only the essentials are summarized below and the EC_50_ values for myr-NCδ in modulating the guanylate cyclase activities of the mutants are provided in Table 3.

ΔH1 mutant: basal activity 20 pmol cGMP min^-1^ (mg prot)^-1^, myr-NCδ-modulated activity, 105 pmol cGMP min^-1^ (mg prot)^-1^ (Figure 8A), 5-fold stimulation (Figure 8B), Hill coefficient 1.40 ± 0.09.

ΔH2 mutant: basal activity 19 pmol cGMP min^-1^ (mg prot)^-1^, myr-NCδ-modulated activity, 78 pmol cGMP min^-1^ (mg prot)^-1^ (Figure 8A), ∼4-fold stimulation (Figure 8B), Hill coefficient 0.87 ± 0.12.

ΔH3 mutant: basal activity 22 pmol cGMP min^-1^ (mg prot)^-1^, myr- NCδ-modulated activity, 109 pmol cGMP min^-1^ (mg prot)^-1^ (Figure 8A), ∼5-fold stimulation (Figure 8B), Hill coefficient 1.23 ± 0.08.

R^802^C mutant: basal activity 24 pmol cGMP min^-1^ (mg prot)^-1^, myr-NCδ-modulated activity, 124 pmol cGMP min^-1^ (mg prot)^-1^ (Figure 8A), ∼5-fold stimulation (Figure 8B), Hill coefficient 1.12 ± 0.11.

ΔH4 mutant: basal activity 105 pmol cGMP min^-1^ (mg prot)^-1^, myr-NCδ-modulated activity, 346 pmol cGMP min^-1^ (mg prot)^-1^ (Figure 8A), ∼3.5-fold stimulation (Figure 8B), Hill coefficient 0.91 ± 0.09. Note: Despite lower x-fold stimulation this mutant’s V_max_ was comparable to that of the wt-ANF-RGC.

ΔH5 mutant: basal activity 9 pmol cGMP min^-1^ (mg prot)^-1^, myr-NCδ-modulated activity, 40 pmol cGMP min^-1^ (mg prot)^-1^ (Figure 8A), ∼4.5-fold stimulation (Figure 8B), Hill coefficient 0.94 ± 0.07.

The above results demonstrate that Ca^2+^/myr-NCδ-dependent activation of ANF-RGC is independent of SHD.

## Discussion

One of the seven members, ANF-RGC is the first discovered membrane guanylate cyclase (Paul, 1986; Paul et al., 1987; Sharma, 1988). This discovery is historic because it established cyclic GMP as the third hormonal second messenger and the membrane guanylate cyclase as a bona fide signal transduction system (Reviewed in Sharma et al., 2016). Structurally, in contrast to the other two, cyclic AMP and IP3, the system is neither G-protein driven nor a three-component signaling system. Instead, and uniquely, it is a single component hormonal (ANF) signal transducer that generates cyclic GMP and also embeds the hormonal receptor. After directly binding the hormone, it accelerates the production of cyclic GMP. Hydropathic analysis of its cloned form reveals ANF-RGC to be a multi-modular protein (Chang et al., 1989; Chinkers et al., 1989; Pandey and Singh, 1990), and the prediction was made that the interplay of these modules is required for the ANF signal transduction (Review Sharma et al., 2016; Sharma and Duda, 1997).

More than four decades-research bears out this prediction. Studies with the reconstructed ANF-RGC gene demonstrated that the ANF-receptor binding site resided in the guanylate cyclase’s ExtD (Duda et al., 1991), and the deletion analysis disclosed that the its ATP-regulated and catalytic site resided in ICD (Marala et al., 1992). These studies began to decode the function-based identity of these modules, define their biochemistry and link them with multiple physiological processes. Thereby, it is now established that ANF-RGC is not only the sole transducer of the hormonal signals, ANF and BNP, but also of the free [Ca^2+^] (Duda et al., 2014).

Empowered with the bimodal characteristic, utilizing entirely different modes, extraordinarily, ANF-RGC is directly linked with the physiological control of blood pressure regulation, hypertension and cardiac hypertrophy (Duda et al., 2012b, 2013; Review: Duda et al., 2014). And, equally amazingly, two signaling pathways, hormonal- and Ca^2+^-modulated never overlap, yet they are translated at a common CCD site. The present study focuses on these issues; identifies the SHD, discloses its three-dimensional model, demonstrates that the two signaling pathways different in their dependencies of on SHD, and then moves on to provide their advanced molecular descriptions.

### Structure and Biochemistry of SHD

The original definition of the SHD was “a long helix consisting of multiple copies of a heptad (7-amino-acid) repeating unit …. with each heptad containing a similar configuration of residues. …The resulting coiled coil may be either parallel or anti-parallel depending on the orientation of the dimerization partners.” (Anantharaman et al., 2006).

Except for STa-RGC (Saha et al., 2009) and ROS-GC1 (Duda et al., 2012b; Zagel et al., 2013), the SHD boundary of any MGC member has not been precisely mapped. The present report demonstrates that it is represented by the amino acid region ^783^N-P^822^ in ANF-RGC. Within it resides its core 35-amino acid α-helical region, ^786^S-I^820^ (Figures 1A–C). Based on the theoretical criterion set forth in Anantharaman et al. (2006), it is composed of five helical heptads, each heptad exhibiting its unique regulatory role in transmission of the ANF/ATP-dependent down-stream signaling pathway for its translation into generation of the cyclic GMP.

Our 3D-model of the SHD is based on the docking results obtained through two programs, M-ZDOCK and Z-DOCK. Up to 30 top ranked M-ZDOCK predicted conformations and 6 out of top-10 ranked Z-DOCK predicted conformations show that in ANF-RGC the most energy-favorable dimeric arrangement of the two helical monomers is antiparallel (Figure 1B). These data form the base for our cautious proposal of ANF-RGC SHD conformation. This type of arrangement is in agreement with the crystallographic results of Ma et al., on the signaling helix coiled-coil domain of rat beta1 subunit of the soluble guanylyl cyclase (Ma et al., 2010) but in disagreement with the electron microscopic results on rat soluble guanylate cyclase coiled-coil (Campbell et al., 2014) and crystallographic, on *Mycobacterium intracellulare* adenylate cyclase (Vercellino et al., 2017). In addition, studies on serine chemotaxis receptor identified the signaling helix consisting of “long coiled coil stretches in both parallel and antiparallel configurations” (Kim et al., 1999).

Is it feasible that the SHD monomers within the ANF-RGC molecule form an antiparallel dimer? We compared the protein sequences of Cya from *Mycobacterium intracellulare* (Vercellino et al., 2017) with rat ANF-RGC (Supplementary Figures S5, S6). Unlike Cya, ANF-RGC is not a multipass transmembrane protein. In ANF-RGC there are more than 300 amino acids between the TM and the SHD domains, while there are only 2 amino acids between TM and SHD regions in Cya. Thus, it makes sense that the parallel arrangement of the Cya SHD monomers is the only possibility. In ANF-RGC, however, considering the spacing between the TM and SHD segments, the dimerizing monomers have the possibility to acquire either a parallel or antiparallel arrangement. Although we appreciate the limitations of the modeling approach, we also realize that the experimental (crystallography or electron microscopy) structure of the ANF-RGC SHD is not known yet, and that the differences in the Spatial arrangement of Secondary structure Elements (SSE) - especially the distance between the transmembrane and SHD segment – between ANF-RGC and adenylate cyclases are significant (*Mycobacterium intracellulare* adenylate cyclase), we regard our results a trigger for further research on the ANF-RGC signal transduction mechanism.

Twelve residues (Figure 1C, indicated by asterisks) form the dimer interphase. Disclosure of this antiparallel feature assumes a significant structural importance because it represents a mode of communication between three-dimensional fold of SHD with the three-dimensional antiparallel fold of the CCD dimer (Liu et al., 1997; Duda et al., 2012b; Ravichandran et al., 2017). Together with the prior evidence that configuration of the ExtD dimer is head-to-head (Ogawa et al., 2004) and that it is the point of origin of the ANF/ATP signal (Duda et al., 1991), it is now concluded that this down-stream signal flows from the parallel-oriented receptor module to the terminal antiparallel-oriented modules of SHD and CCD for translation into the generation of cyclic GMP.

Through site-directed mutational and expression analysis of the detergent-exposed mutants of STa-RGC (Saha et al., 2009), a common functional theme of the SHD structure in the down-stream signaling path has been proposed. Here, SHD interacts with “regions on the guanylate cyclase domain, thereby acting as a clamp to ensure low levels of cyclic GMP production.” This means that SHD is a constitutive switch; it suppresses the basal catalytic activity of CCD in its native state and releases the suppression to the downward flowing hormonal signal, causing amplification of the CCD activity and generating accelerated production of the cyclic GMP.

Our previous study with ROS-GC1 (Duda et al., 2012c) and the present one with ANF-RGC supports the general conclusion that SHD is a critical transmission switch of the down-stream signaling pathways, yet it disputes the concept that it is a general suppressor of the basal CCD activity (Saha et al., 2009). We conclude this through analysis of the deleted SHD module of ANF-RGC. This mutant ( ΔSHD) and the wt-ANF-RGC have almost identical basal catalytic activities. Thus, in its native state SHD is not the suppressor of the CCD’s activity. Rather, in accord with the previous findings (Hirayama et al., 1993; van den Akker et al., 2000; Venkataraman et al., 2008; Saha et al., 2009; Zagel et al., 2013; Ravichandran et al., 2017), the present study demonstrates that the SHD and CCD are operationally independent modules, the latter controlling its own intrinsic catalytic activity.

Importantly, these results further challenge the alternate concept that the SHD in ANF-RGC (termed DD by the investigators) (Garbers, 1992; Wilson and Chinkers, 1995) and ROS-GC1 (Tucker et al., 1999; Ramamurthy et al., 2001; Peshenko et al., 2004; Dizhoor et al., 2016) is indispensable for dimerization of the CCD. The CCD exists as a functional homo-dimer in the absence of SHD (Venkataraman et al., 2008; Duda et al., 2012c). Additionally, circular dichroism experiments demonstrate that the isolated SHD in STa-RGC and in ROS-GC1 does not form a typical coiled-coil (Saha et al., 2009; Zagel et al., 2013).

#### Five-Heptad Intra-Helical-Regions Control Basal Catalytic Activity of ANF-RGC Through Differential Modes

Although intact SHD has no role in controlling the basal catalytic activity of the CCD, analysis of the individual heptads demonstrates surprising results. Every heptad individually controls the CCD’s catalytic activity. And, except for H4, the pattern is that they all amplify it, H5 being the most effective. H4 suppresses it. Additionally, like its parent H3, the arginine, R^802^, residue in it also amplifies the basal catalytic activity of ANF-RGC.

To explain these mechanisms, important clues are provided by the kinetic properties of these deleted heptads (Table 2). The deletions or point mutation in H3 do not affect K_M_ values for the substrate GTP, yet affect catalytic efficiency (Table 2). Thus, individually the heptads are connected with the 7-residue catalytic element of the CCD. In this manner, they control the wreath- or circlet-like configuration of the element, and degree of its catalytic activity (Ravichandran et al., 2017). However, collectively, under native conditions they compensate each other’s affect to keep the configuration of the catalytic center optimal for its normal catalytic operation. Yet, if their structural alignment in the core SHD is disturbed, e.g., by mutation, they concomitantly destabilize the CCD and affect its basal catalytic activity. This is illustrated by the fact that site-directed mutations in H4 of the ROS-GC1 cause significant rise in the basal catalytic activities of the mutants (Zagel et al., 2013), consistent with the present results established for the properties of ΔH4 mutant.

#### Heptad 3 and Its Conserved R Residue

In ANF-RGC similar to ROS-GC1, heptad 3 of the SHD and its node “R” residue are critical in tuning total modular activity of the cyclase. The R residue is the central part of the “ERT” conserved signature motif in the entire membrane guanylate cyclase family (Anantharaman et al., 2006) and its naturally occurring mutations cause a loss of function and lead to a serious abnormalities (Vasques et al., 2013; Nakao et al., 2015; Sharon et al., 2017; Duda et al., 2018). The most frequently observed is the R to C substitution. Why it has so severe consequences? They can be explained by the profound differences in biochemical properties of these two aminoacids (data from Amino Acid Explorer)^6^. The R residue has high side-chain flexibility, has the ability to form ionic and up to 7 hydrogen bonds, its isoelectric point is 10.8, and it is hydrophilic; in contrast, the C residue has low side chain flexibility, forms mostly covalent disulphide bonds, and only under certain conditions can form hydrogen bonds (Mazmanian et al., 2016; van Bergen et al., 2016) but never ionic bonds; its isoelectric point is 5, and hydrophobicity is 0.721. Most importantly, the R to C substitution is extremely rare as reflected by its BLOSUM62 score (-3).

### SHD Is the ANF/ATP Signal Transmission Switch to CCD

Early studies demonstrate that ROS-GC1 transduces two Ca^2+^-sensor-modulated, GCAP1 and GCAP2, pathways (Duda et al., 2012c). The trajectories of these pathways are opposite, GCAP1’s down-stream and GCAP2’s upstream. Only in the down-stream pathway SHD constitutes the pathway TRANSMISSION SWITCH to the CCD. Does SHD also constitute the signaling switch for the down-stream ANF/ATP signaling pathway?

The answer is yes. The SHD-deleted mutant of ANF-RGC does not respond to the ANF/ATP signaling. Thus, the SHD switch is the gateway for the down-stream ANF/ATP signaling pathway. We propose that this may constitute a common theme of all the down-stream signaling pathways.

The conclusions on the SHD role in ANF-RGC signaling are in a general agreement with the available results on the role of the signaling helix in activation of the receptor/adenylyl cyclase reporter Artrospira maxima (Winkler et al., 2012). Deletion of various fragments of the SHD resulted in an increase or decrease of the enzyme basal activity and reversal or lack of response to its ligand, serine, signaling.

Incorporating this new feature, we now advance our existing model (Duda et al., 2013) of “*ANF/ATP Signaling of ANF-RGC, Cyclic GMP Production Events.”* Barring this addition, the model narration remains unchanged.

**FIGURE 9:**
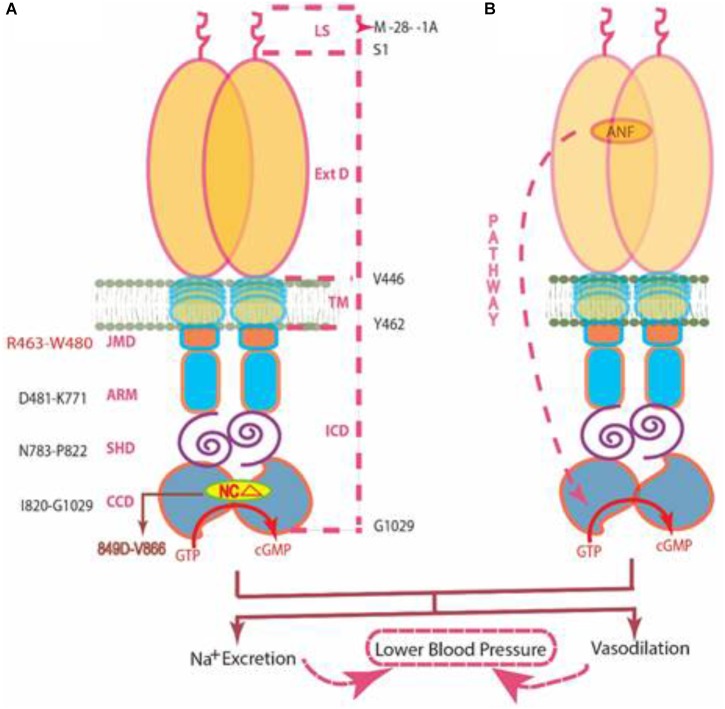
Two independent signaling pathways of ANF-RGC activity. **(A)** Ca^2+^/myr-NCδ. ANF-RGC is a single-transmembrane spanning homodimer protein. The dashed lines at the right show the defined boundaries of its segments: LS, leader sequence; ExtD, extracellular domain; TM, transmembrane domain; ICD, intracellular domain. The functional domains housed in ICD are: JMD, juxtamembrane domain; ARM, ATP regulated module; SHD, signaling helix domain; CCD, core catalytic domain. The site targeted by neurocalcin δ (NCΔ) (encircled) is located within the CCD. Thus, the Ca^2+^/myr-NCδ signal originates and is transduced at the CCD. **(B)** ANF/ATP. The trajectory of ANF signaling pathway is shown with a red dashed arrow. From its origin at the ExtD, it passes through the structural domains of TM, ARM (ATP binds to ARM in response to ANF signal), and SHD on its way to CCD. The figure was modified from (Duda et al., 2012b).

MODEL (Figure 9): The signal originates by binding of one molecule of ANF to the ExtD of the ANF-RGC dimer (Duda et al., 1991; van den Akker et al., 2000). The binding modifies the juxta-membrane domain where the disulfide ^423^C-C^432^ structural motif is a key element (Ogawa et al., 2004; Duda and Sharma, 2005). The signal twists the TMD, induces a structural change in the ARM domain, and prepares it for the ATP binding (Burczynska et al., 2007). Step 1, the ARM domain binds ATP, which leads to a cascade of temporal and spatial changes (Duda et al., 2000b). They involve a shift in the ATP binding pocket position by 3–4 Å and rotation of its floor by 15° (G^505^ acts as a critical pivot for both the shift and the rotation), (2) movement by 2–7 Å but not the rotation of its β4 and β5 strands and its loop, and (3) movement of its αEF helix by 2–5 Å. This movement exposes the hydrophobic motif, ^669^WTAPELL^675^, which through SHD facilitates its direct interaction with the CCD resulting in its partial, ∼50%, activation (Duda et al., 2009). Step 2, the six phosphorylation sites are brought from their buried state to their exposed state (Duda et al., 2011b). ATP, through a hypothetical protein kinase, phosphorylates the residues, and full activation (additional 50%) of ANF-RGC is achieved. ANF-dependent cyclic GMP is generated and functions as the second messenger of blood pressure regulation. Concomitantly, phosphorylation converts the ATP binding site from high to low affinity; ATP dissociates, and ANF-RGC returns to its ground state 55 (Duda et al., 2011b).

### SHD Has No Role in Transmission of the Ca^2+^-Modulated ANF-RGC Signaling Pathway

In contrast to the ANF/ATP-modulated ANF-RGC signaling pathway, the myr-NCδ-modulated Ca^2+^ signaling pathway is main-stream, it originates and is translated in the CCD (Duda et al., 2014). In addition to the SHD, its other N-terminal modules - - ExtD, JMD and ARM- - have no role in transmission of the Ca^2+^-modulated pathway to CCD (Figures 7, 8). In this manner, this pathway does not overlap with the ANF/ATP-modulated pathway; consequently the two pathways operate independently. This transduction model “*Ca^2^*^+^/*myr-NC*d *signaling of ANF-RGC*” is illustrated below. [Note, studies with ROS-GC1 have demonstrated that the conserved WTAPELL motif constitutes hinge region of the membrane guanylate cyclase and is critical for both downstream and upstream signaling pathways (Duda et al., 2011a); it is therefore possible that this may be a general property of the MGC family].

MODEL (Figure 9): The CCD is composed of three subdomains. Between the two flanking, N-terminal and C-terminal, resides the third ^849^DIVGFTALSAESTPMQVV^866^ conserved subdomain of the MGC family (Ravichandran et al., 2017). Notably, it contains none of the residues forming the core catalytic element; all seven residues constituting the element reside in the two flanking domains. This domain represents the universal NCδ binding site. The active form of NCδ is its myristoylated dimeric form (Duda et al., 2012b). The domain becomes functional upon binding Ca^2+^-bound myr-NCδ; and it functions by activating the seven-residue catalytic element that spans the two flanking CCD subdomains (Ravichandran et al., 2017).

In the basal state, one dimer of myr-NCδ is bound to its target site in the CCD (Duda et al., 2012b). The catalytic activity of ANF-RGC is at the threshold level in the production of cyclic GMP from GTP. In the activated state, an increase in free [Ca^2+^]_i_ with K_1/2_ of 0.5 μM is captured by the myr-NCδ; It undergoes Ca^2+^-dependent configurational change/s, this, in turn, causes a concomitant structural change in the CCD’s catalytic center. The residues, seven from each chain, align to form a wreath-like structure, it enhances its catalytic efficiency (*k_cat_*) and generates the saturated production of cyclic GMP.

It needs to be stressed again that the outstanding feature of this bimodal operation is that both operations occur independently, their operational modes are totally different, their signaling pathways never overlap, they energize the CCD through different mechanisms, yet they converge to control the common processes of blood and cardio-vasculature regulation (Duda et al., 2012a, 2013).

## Conclusion

This study represents a continuation of the saga on the development of the membrane guanylate cyclase (MGC) field and is a part of the overall goal of decoding the molecular principles governing the transduction machineries of various MGCs. About five-decades ago the existence of hormonally regulated MGC was negated. Studies by the authors’ group on the epinephrine-sensitive rat adrenal 494 carcinoma cells, challenged this notion (Perchellet and Sharma, 1980; Shanker and Sharma, 1980; reviewed in Sharma, 2002). Meticulous dissection of the MGC system elevated its stature from anonymity to being a preeminent signal transducer that generates intracellular second messenger, cyclic GMP and controls countless physiological processes.

Now, we focus on two signals that modulate the catalytic activity of ANF-RGC in the physiology of blood pressure regulation: one, hormonal ANF/ATP and the other, non-hormonal Ca^2+^, signaling through its sensor myr-NCδ. These signals employ different modes of regulating ANF-RGC catalytic activity. The ANF/ATP signal originating at the extracellular portion of the cyclase is critically dependent on the integrity of the signaling helix, whereas the Ca^2+^ signal is independent of it. Finally, using the appropriate molecular modeling tools we show that the most plausible configuration of the ANF-RGC signaling helix is an antiparallel or head-to-tail dimer.

## Author Contributions

TD designed, carried out the experiments, and analyzed the results. SR created the 3D models. AP created and expressed all the mutants. RS conceptually planned and coordinated the study. All authors contributed to the writing of the manuscript.

## Conflict of Interest Statement

The authors declare that the research was conducted in the absence of any commercial or financial relationships that could be construed as a potential conflict of interest.

## Footnotes

1 http://zhanglab.ccmb.med.umich.edu/I-TASSER/

2 http://zdock.umassmed.edu/m-zdock

3 http://zdock.umassmed.edu/

4 http://coiledcoils.chm.bris.ac.uk/socket

5 http://zhanglab.ccmb.med.umich.edu/PSSpred/

6 https://www.ncbi.nlm.nih.gov/Class/Structure/aa/aa_explorer.cgi
