# Supplementary material for: Ca2+-Sensor Neurocalcin δ and Hormone ANF Modulate ANF-RGC Activity by Diverse Pathways: Role of the Signaling Helix Domain
Source: Front Mol Neurosci. 2018 Nov 27;11:430. doi: 10.3389/fnmol.2018.00430 (PMC6278801; doi:10.3389/fnmol.2018.00430)
Supplement: Supplementary file 1 [file Data_Sheet_1.pdf]

Supplementary Information

Figure S1: Secondary structure prediction by PSSpred (I-TASSER pipeline software; http://zhanglab.ccmb.med.umich.edu/PSSpred/)

```
                                        20                   40
                                         |                    |
Sequence      NLLSRMEQYANNLEELVEERTQAYLEEKRKAEALLYQILP
Prediction    CHHHHHHHHHHHHHHHHHHHHHHHHHHHHHHHHHHHHHCC
Conf.Score    9787999999778999999999999999999999974194

H:Helix; S:Strand; C:Coil
```

Figure S2. Schematic diagram detailing the ANF-RGC signaling helix modeling, dimer structure predictions and analysis.

NLLSRMEQYANNLEELVEERTQAYLEEKRKAEALLYQILP

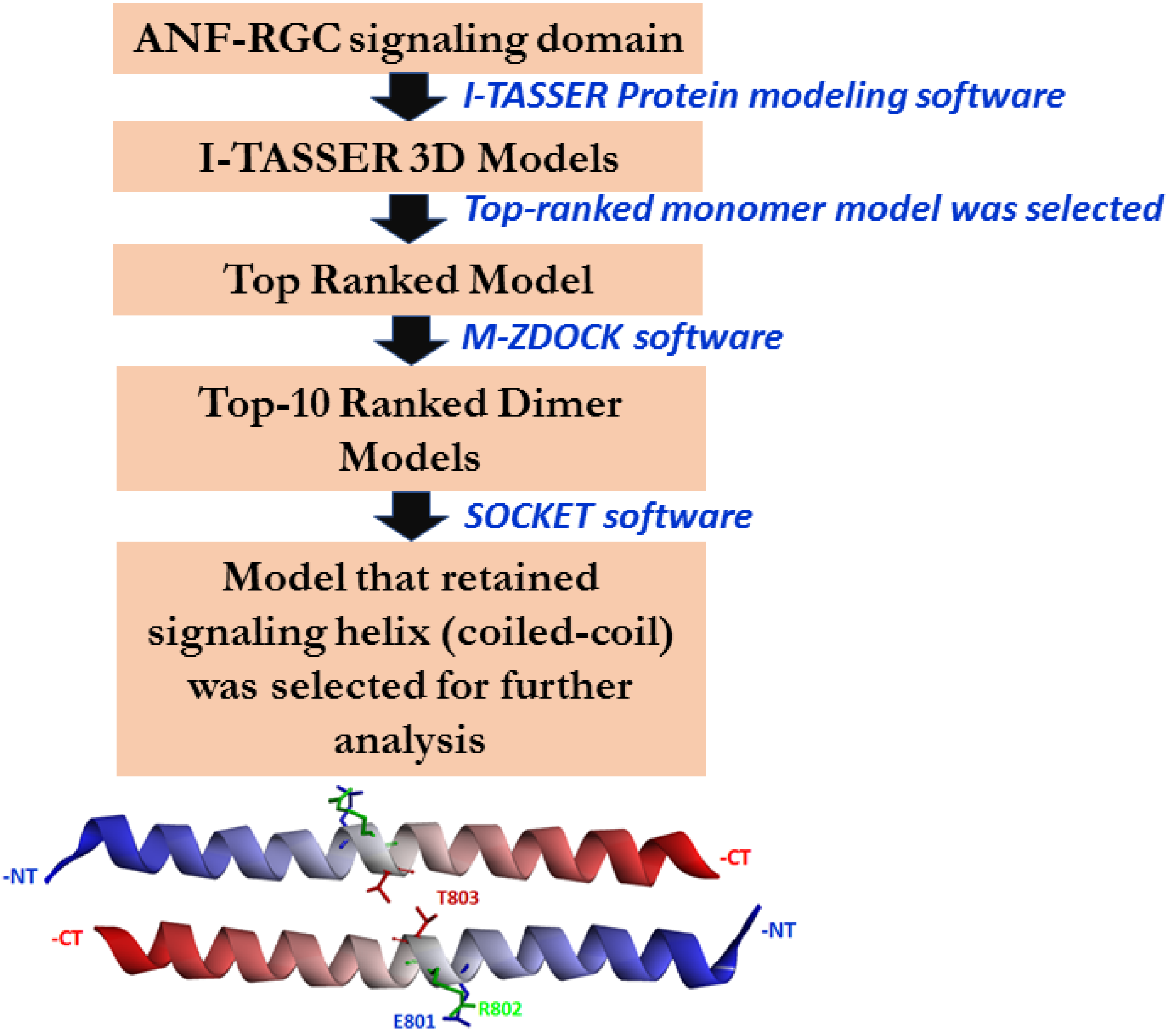

Figure S3: Top ten M-ZDOCK predicted homodimer complexes. Protein complexes are displayed in ribbon style and colored by N-to-C scheme where the N-terminal end is colored blue and the C-terminal end in red, and the intermediate region is colored by a continuous gradient from blue to red.

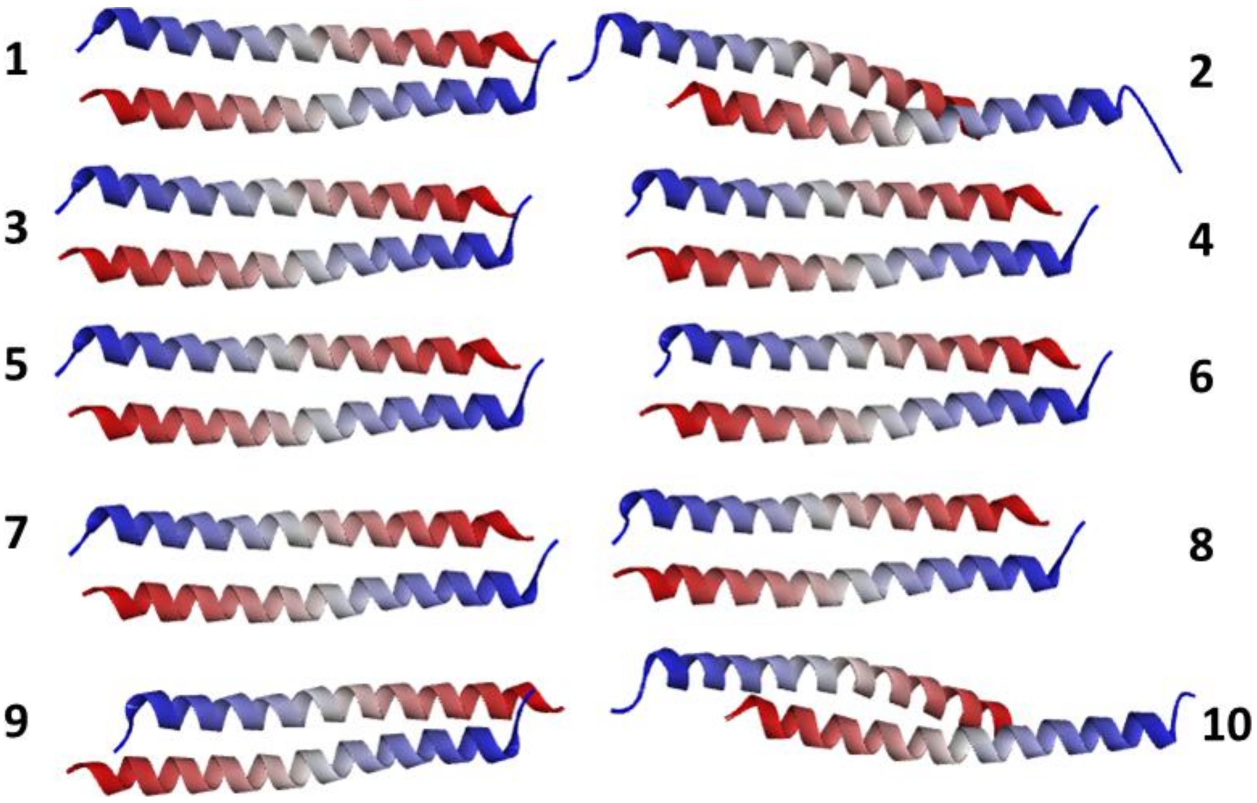

Figure S4: SOCKET prediction for M-ZDOCK complex (rank 4 complex, complex.4.pdb)

SOCKET v3.02 02-11-01 John Walshaw, University of Sussex

using cutoff of 7.0 Angstroms for centre of mass distances

attempting to open "/tmp/tmpJFOFXN//complex.4.pdb"

opened "/tmp/tmpJFOFXN//complex.4.pdb" (input)

opened "/tmp/tmpJFOFXN//complex.4.dssp" (input)

opened "/tmp/tmpJFOFXN//complex.4.7.0.long.socket" (output)

Found beginning of residue data

There are 70 alpha-helical residues in this structure

chain A starts at residue 1, iCode=' '

chain B starts at residue 1, iCode=' '

These are the knobs and holes:

knobs in helix 0:

0) 10 (GLU 14:A, iCode=' ', helix 0) type 4 (hole: LYS 28:B iCode=' ', ALA 31:B iCode=' ', GLU 32:B iCode=' ', LEU 35:B iCode=' ' helix 1) packing angle 80.546

1) 14 (GLU 18:A, iCode=' ', helix 0) type 3 (hole: LEU 25:B iCode=' ', LYS 28:B iCode=' ', ARG 29:B iCode=' ', GLU 32:B iCode=' ' helix 1) packing angle 172.368

2) 24 (LYS 28:A, iCode=' ', helix 0) type 4 (hole: GLU 14:B iCode=' ', VAL 17:B iCode=' ', GLU 18:B iCode=' ', THR 21:B iCode=' ' helix 1) packing angle 95.891

3) 28 (GLU 32:A, iCode=' ', helix 0) type 4 (hole: ASN 11:B iCode=' ', GLU 14:B iCode=' ', GLU 15:B iCode=' ', GLU 18:B iCode=' ' helix 1) packing angle 159.857

knobs in helix 1:

4) 45 (GLU 14:B, iCode=' ', helix 1) type 4 (hole: LYS 28:A iCode=' ', ALA 31:A iCode=' ', GLU 32:A iCode=' ', LEU 35:A iCode=' ' helix 0) packing angle 80.546

5) 49 (GLU 18:B, iCode=' ', helix 1) type 3 (hole: LEU 25:A iCode=' ', LYS 28:A iCode=' ', ARG 29:A iCode=' ', GLU 32:A iCode=' ' helix 0) packing angle 172.368

6) 59 (LYS 28:B, iCode=' ', helix 1) type 4 (hole: GLU 14:A iCode=' ', VAL 17:A iCode=' ', GLU 18:A iCode=' ', THR 21:A iCode=' ' helix 0) packing angle 95.891

7) 63 (GLU 32:B, iCode=' ', helix 1) type 4 (hole: ASN 11:A iCode=' ', GLU 14:A iCode=' ', GLU 15:A iCode=' ', GLU 18:A iCode=' ' helix 0) packing angle 159.857

holes in helix 0:

LYS 28:A iCode=' ', ALA 31:A iCode=' ', GLU 32:A iCode=' ', LEU 35:A iCode=' ' (knob: 45 (GLU 14:B, helix 1))

LEU 25:A iCode=' ', LYS 28:A iCode=' ', ARG 29:A iCode=' ', GLU 32:A iCode=' ' (knob: 49 (GLU 18:B, helix 1))

GLU 14:A iCode=' ', VAL 17:A iCode=' ', GLU 18:A iCode=' ', THR 21:A iCode=' ' (knob: 59 (LYS 28:B, helix 1))

ASN 11:A iCode=' ', GLU 14:A iCode=' ', GLU 15:A iCode=' ', GLU 18:A iCode=' ' (knob: 63 (GLU 32:B, helix 1))

holes in helix 1:

LYS 28:B iCode=' ', ALA 31:B iCode=' ', GLU 32:B iCode=' ', LEU 35:B iCode=' ' (knob: 10 (GLU 14:A, helix 0))

LEU 25:B iCode=' ', LYS 28:B iCode=' ', ARG 29:B iCode=' ', GLU 32:B iCode=' ' (knob: 14 (GLU 18:A, helix 0))

GLU 14:B iCode=' ', VAL 17:B iCode=' ', GLU 18:B iCode=' ', THR 21:B iCode=' ' (knob: 24 (LYS 28:A, helix 0))

ASN 11:B iCode=' ', GLU 14:B iCode=' ', GLU 15:B iCode=' ', GLU 18:B iCode=' ' (knob: 28 (GLU 32:A, helix 0))

knob 0 (residue 10 = GLU 14:A iCode=' ') type 4 order 2

knob 1 (residue 14 = GLU 18:A iCode=' ') type 3 order 2

knob 2 (residue 24 = LYS 28:A iCode=' ') type 4 order 2

knob 3 (residue 28 = GLU 32:A iCode=' ') type 4 order 2

knob 4 (residue 45 = GLU 14:B iCode=' ') type 4 order 2

knob 5 (residue 49 = GLU 18:B iCode=' ') type 3 order 2

knob 6 (residue 59 = LYS 28:B iCode=' ') type 4 order 2

knob 7 (residue 63 = GLU 32:B iCode=' ') type 4 order 2

coiled coil 0: 2 helices 0 1 frequency 4

helix 0 is in a 2-stranded coiled coil

helix 1 is in a 2-stranded coiled coil

coiled coil 0:

angle between helices 0 and 1 is 179.511 antiparallel

this coiled coil is antiparallel

complex.4.pdb 7.0 0 coiled coil (i) 0 (antiparallel 2-stranded, length max 25 mean 25.00):

assigning heptad to helix 0 (X) 4-38:A

extent of coiled coil packing: 11- 35:A

sequence SRMEQYANNLEELVEERTQAYLEEKRKAEALLYQI

register abcdefgabcdefgabcdefgabcd

partner ----------Y---Y---------Y---Y------

knobtype ----------4---3---------4---4------

repeats 0 non-canonical interrupts in 25 residues: 7,7,7,4

assigning heptad to helix 1 (Y) 4-38:B

extent of coiled coil packing: 11- 35:B

sequence SRMEQYANNLEELVEERTQAYLEEKRKAEALLYQI

register abcdefgabcdefgabcdefgabcd

partner ----------X---X---------X---X------

knobtype ----------4---3---------4---4------

repeats 0 non-canonical interrupts in 25 residues: 7,7,7,4

complex.4.pdb c 7.00 e 0 result 1 COILED COILS PRESENT

To explain the possibility of antiparallel configuration for the ANF-RGC SHD), the sequence of the cya from the Mycobacterium intracellulare 1956 (Vercellino et al., 2017) (Fig. S5) was compared with the sequence of rat ANF-RGC (Fig. S6). The TM segments are highlighted in green and the SHD segments in yellow.

Figure S5: Protein sequence of Cya. Transmembrane segments are highlighted in green and the signaling helix, SHD, in yellow. Transmembrane feature locations were extracted from UniProtKB database (https://www.uniprot.org/uniprot/X8CHM4#sequences).

```
                              10        20        30        40        50        60
tr|X8CHM4|X8CHM4_MYCIT  MAAKDCGGPPRWPDGSSKRPDCVAAARAQSRARNQHYADSAARQYRVLAIAAWLAVLVCV
                              70        80        90       100       110       120
tr|X8CHM4|X8CHM4_MYCIT  DFVALQLITGVWTWQIISLNALAAMIFAAVPWLHRFGDLIAPLTFIGAAYVTVFVSTWDA
                             130       140       150       160       170       180
tr|X8CHM4|X8CHM4_MYCIT  GTGTGAQFFFLVGACLVVLLLGIEHTVLAAGLTAVGAGLVIALEFLVPRDTGLQPAWAQS
                             190       200       210       220       230       240
tr|X8CHM4|X8CHM4_MYCIT  MTFVVTIVSACVMVFVAVWFALRDTQRAEAVMEAEYERSEALLANMLPGSIAERLKSSSR
                             250       260       270       280       290       300
tr|X8CHM4|X8CHM4_MYCIT  SVIADKYDEVSVLFADIVGFTERASTTTPADLVRFLNRLYGAFDELVDKHGLEKIKVSGD
                             310       320       330       340       350       360
tr|X8CHM4|X8CHM4_MYCIT  SYMVVSGVPRARPDHAFALADFALDMANVAAALKDPHGDPVALRMGMACGPVVAGVVGSR
                             370       380       390       400       410       420
tr|X8CHM4|X8CHM4_MYCIT  RFFYDVWGDAVNVASRMESTDSVGRIQVPEAMYERLKNEFVLQERGRIEVKGKGVMRTWY
                             430       440       450       460       470       480
tr|X8CHM4|X8CHM4_MYCIT  LIGRKADEEPADLLTGRPHTVHV
```

Figure S6: Protein sequence of ANPRA_RAT. Transmembrane segment is highlighted in green and the signaling helix, SHD, in yellow. Transmembrane location was extracted from https://www.uniprot.org/uniprot/P18910

sp|P18910|ANPRA_RAT	MPGSRRVRPRLRALLLLPPLLLLRGGHASDLTVAVVLPLTNTSYPWSWARVGPAVELALA	60
sp|P18910|ANPRA_RAT	RVKARPDLLPGWTVRMVLGSSENAAGVCSDTAAPLAAVDLKWEHSPAVFLGPGCVYSAAP	120
sp|P18910|ANPRA_RAT	VGRFTAHWRVPLLTAGAPALGIGVKDEYALTTRTGPSHVKLGDFVTALHRRLGWEHQALV	180
sp|P18910|ANPRA_RAT	LYADRLGDDRPCFFIVEGLYMRVRERLNITVNHQEFVEGDPDHYPKLLRAVRRKGRVIYI	240
sp|P18910|ANPRA_RAT	CSSPDAFRNLMLLALNAGLTGEDYVFFHLDVFGQSLKSAQGLVPQKPWERGDGQDRSARQ	300
sp|P18910|ANPRA_RAT	AFQAAKIITYKEPDNPEYLEFLKQLKLLADKKFNFTVEDGLKNIIPASFHDGLLLYVQAV	360
sp|P18910|ANPRA_RAT	TETLAQGGTVTDGENITQRMWNRSFQGVTGYLKIDRNGDRDTDFSLWDMDPETGAFRVVL	420
sp|P18910|ANPRA_RAT	NYNGTSQELMAVSEHKLYWPLGYPPPDVPKCGFDNEDPACNQDHFSTLEVLALVGSLSLI	480
sp|P18910|ANPRA_RAT	SFLIVSFFIYRKMQLEKELVSELWRVRWEDLQPSSLERHLRSAGSRLTLSGRGSNYGSLL	540
sp|P18910|ANPRA_RAT	TTEGQFQVFAKTAYYKGNLVAVKRVNRKRIELTRKVLFELKHMRDVQNEHLTRFVGACTD	600
sp|P18910|ANPRA_RAT	PPNICILTEYCPRGSLQDILENESITLDWMFRYSLTNDIVKGMLFLHNGAICSHGNLKSS	660
sp|P18910|ANPRA_RAT	NCVVDGRFVLKITDYGLESFRDPEPEQGHTLFAKKLWTAPELLRMASPPARGSQAGDVYS	720
sp|P18910|ANPRA_RAT	FGIILQEIALRSGVFYVEGLDLSPKEIIERVTRGEQPPFRPSMDLQSHLEELGQLMQRCW	780
sp|P18910|ANPRA_RAT	AEDPQERPPFQQIRLALRKFNKENSSNILDNLLSRMEQYANNLEELVEERTQAYLEEKRK	840
sp|P18910|ANPRA_RAT	AEALLYQILPHSVAEQLKRGETVQAEAFDSVTIYFSDIVGFTALSAESTPMQVVTLLNDL	900
sp|P18910|ANPRA_RAT	YTCFDAVIDNFDVYKVETIGDAYMVVSGLPVRNGQLHAREVARMALALLDAVRSFRIRHR	960
sp|P18910|ANPRA_RAT	PQEQLRLRIGIHTGPVCAGVVGLKMPRYCLFGDTVNTASRMESNGEALKIHLSSETKAVL	1,020
sp|P18910|ANPRA_RAT	EEFDGFELELRGDVEMKGKGKVRTYWLLGERGCSTRG	1,080

Unlike Cya, ANF-RGC is not a multipass transmembrane protein. There are more than 300 amino acids between the transmembrane motif and the signaling helix. Given the fact that there are 2 amino acids between TM domain and the signaling helix in Cya, it makes sense that the parallel arrangement of signaling helices in dimers is the only possibility. In ANF-RGC, considering the spacing between the TM and SHD domains, the dimerizing monomers have the possibility to acquire either a parallel or antiparallel arrangement.
